# Loneliness and Risk of Incident Hearing Loss: The UK Biobank Study

**DOI:** 10.34133/hds.0281

**Published:** 2025-05-02

**Authors:** Yunlong Song, Andrew Steptoe, Honghao Yang, Zheng Ma, Lizhi Guo, Bin Yu, Yang Xia

**Affiliations:** ^1^ Institute of Applied Psychology, Tianjin University, Tianjin, China.; ^2^ Department of Behavioural Science and Health, University College London, London, UK.; ^3^ Liaoning Key Laboratory of Precision Medical Research on Major Chronic Disease, Shenyang, China.; ^4^ Department of Clinical Epidemiology, Shengjing Hospital of China Medical University, Shenyang, China.; ^5^ Department of Psychology, The Chinese University of Hong Kong, Shatin, Hong Kong.; ^6^ Academy of Medical Engineering and Translational Medicine, Tianjin University, Tianjin, China.; ^7^ School of Public Health, Shenyang Medical College, Shenyang, China.

## Abstract

**Background:** Hearing loss (HL) is one major cause of disability and can lead to social impairments. However, the relationship between loneliness and the risk of incident HL remains unclear. Our study aimed to investigate this association among adults in the UK. **Methods:** This cohort study was based on data from the UK Biobank study. Loneliness was assessed by asking participants if they often felt lonely. Incident HL was defined as a primary diagnosis, ascertained via linkage to electronic health records. Cox proportional hazard regression models were used to examine the association between loneliness and risk of incident HL. **Results:** Our analyses included 490,865 participants [mean (SD) age, 56.5 (8.1) years; 54.4% female], among whom 90,893 (18.5%) reported feeling lonely at baseline. Over a median follow-up period of 12.3 years (interquartile range, 11.3 to 13.1), 11,596 participants were diagnosed with incident HL. Compared to non-lonely participants, lonely individuals exhibited an increased risk of HL [hazard ratio (HR), 1.36; 95% confidence interval (CI), 1.30 to 1.43]. This association remained (HR, 1.24; 95% CI, 1.17 to 1.31) after adjusting for potential confounders, including age, sex, socioeconomic status, biological and lifestyle factors, social isolation, depression, chronic diseases, use of ototoxic drugs, and genetic risk of HL. The joint analysis showed that loneliness was significantly associated with an increased risk of incident HL across all levels of genetic risks for HL. **Conclusions:** Loneliness was associated with the risk of incident HL independent of other prominent risk factors. Social enhancement strategies aimed at alleviating loneliness may prove beneficial in HL prevention.

## Introduction

Hearing loss (HL) refers to a partial or complete inability to apprehend sound, typically characterized by an elevation in the auditory threshold relative to the average hearing threshold [1]. This sensory impairment has been a global public health challenge, affecting more than 1.5 billion people worldwide [2], with substantial personal and societal burdens [3]. As a major contributor to years lived with disability [4], HL has been associated with various adverse health conditions, such as depression [5], cognitive decline [6], impaired physical function [7], and an increased risk of cardiovascular disease (CVD) [8]. Moreover, HL is regarded as an important modifiable risk factor for dementia [9]. Given the limited efficacy of current therapeutic strategies for HL, it is becoming increasingly crucial to focus on prevention through the identification of risk factors [10].

Although physiological (e.g., obesity, diabetes mellitus, and hypertension) and lifestyle factors (e.g., smoking and physical activity) contributing to HL have been identified [11–15], the role of psychosocial factors is less understood. Loneliness, as a mental phenomenon, refers to a subjective feeling of distress, arising from the discrepancy between desired and actual social relationships [16]. Accumulating evidence has shown that loneliness is associated with both mortality and various other adverse health outcomes, particularly among older people [17,18]. Recognizing its growing impact, the World Health Organization has prioritized loneliness as a pressing health threat, establishing a new commission to address the issue on a global scale [19]. Of note, loneliness could lead to an increased risk of CVD [20], with its risk factors largely overlapping those associated with HL [10]. However, to our knowledge, no investigation has explored whether loneliness is linked to the risk of HL.

Previous research has identified the relationship between HL and loneliness, primarily focusing on the perspective that HL leads to increased loneliness due to the frustration caused by the disability to communicate effectively [21,22]. However, this relationship could be bidirectional—loneliness could also lead to an increased risk of developing HL. Although direct evidence connecting loneliness to a higher incidence of HL is limited, existing studies have highlighted several pathways through which loneliness may contribute to auditory system deterioration and the onset of hearing impairment. For example, loneliness has been associated with the development of chronic diseases including hypertension and diabetes [23,24], as well as adverse mental health outcomes like depression [25], all of which have been linked to compromised auditory health. Additionally, lonely individuals are more likely to engage in unhealthy behaviors, such as smoking and physical inactivity, compared to their non-lonely counterparts [26], which may further exacerbate the risk of hearing decline. Physiological pathways may also play a crucial role. Loneliness has been associated with alterations in surrogate biomarkers, including elevated inflammation, increased blood pressure, and maladaptive autonomic stress responses [24,27,28]. These physiological changes can potentially accelerate the onset and progression of hearing impairment by negatively affecting vascular and neural functions critical to auditory health.

Through the mechanisms mentioned above, loneliness may serve as an independent risk factor for HL. Thus, exploring whether loneliness predicts new-onset HL is crucial not only for clarifying the potential bidirectional nature of this relationship but also for developing early prevention strategies. Given the limited research in this field, the current study aimed to investigate the association between loneliness and incident HL in a large cohort of adults living in England. Furthermore, recognizing that HL development is influenced by both environmental and genetic factors [29], we also examined whether genetic risk moderates the relationship between loneliness and HL.

## Methods

### Participants

The UK Biobank is a large population-based cohort study that enrolled over half a million community-dwelling adults at 22 assessment centers across the United Kingdom. The baseline data were collected between 2006 and 2010. During this period, participants completed a comprehensive questionnaire that covered sociodemographic information, health behavior, and medical history. All participants gave their written informed consent. Further details of the study design can be found elsewhere [30]. Ethics approval for data use of UK Biobank was obtained from the North West Multi-centre Research Ethics Committee (21/NW/0157). All participants provided written informed consent for using their data for health-related research purposes. The present study was conducted under application number 63454 of the UK Biobank resource.

### Measures and outcome ascertainment

#### Loneliness

In the UK Biobank, loneliness was assessed with the question: “Do you often feel lonely?” (no = 0; yes = 1). This single-item measure has demonstrated a strong correlation with the multi-item UCLA loneliness scale, one of the most widely used measurements of loneliness [31].

#### Hearing loss

Diagnosis of HL was obtained from hospital inpatient records using the International Classification of Diseases 10th revision codes (ICD-10). Incident cases were identified at the time of the first hospital contact with a main or contributory diagnosis of HL (ICD-10: H90 to H91) [32]. In addition to unspecified HL, subtypes were classified as conductive, sensorineural, and mixed HL. Conductive HL (H90.1 to H90.2) is caused by impairment of the outer or middle ear, preventing sound transmission to the inner ear. Sensorineural HL (H90.3 to H90.5) results from damage to the cochlea or spiral ganglion, affecting the neural pathways involved in hearing [10]. Mixed HL (H90.6 to H90.8) involves both conductive and sensorineural components. Follow-up time was calculated from the baseline date to the diagnosis of the outcome, death, or the censoring date (2021 September 30 for centers in England, 2018 February 28 for centers in Wales, and 2021 July 31 for centers in Scotland), whichever occurred first.

#### Genetic risk score with HL

The genetic predisposition for HL was developed by a genetic risk score for HL using 48 single-nucleotide polymorphisms (SNPs) (Table S1) [29,33]. Each SNP was coded as 0, 1, or 2 based on the number of risk alleles and weighted by the relative effect size (β coefficient). Subsequently, a sum was calculated using the PLINK “–score” command and then z-standardized to generate a genetic risk score for each individual. A higher score indicated a greater genetic predisposition to HL. The genetic risk score was then categorized into low (quintile 1), intermediate (quintiles 2 to 4), and high (quintile 5) for subsequent analyses [34].

#### Covariates

In addition to age, sex, and ethnicity (white versus other), the following variables were included as covariates based on previous studies: socioeconomic factors including education (low, no secondary education; intermediate, secondary education; high, university degree), annual household income (<£31,000 versus ≥£31,000), and the Townsend deprivation index. The Townsend deprivation index is a comprehensive measure used at the neighborhood level across the UK. It takes into account factors such as unemployment, non-car ownership, non-home ownership, and household overcrowding [35].

For behavioral factors, participants self-reported their smoking status (current, ex-, or never smokers), alcohol intake (≥3 versus <3 times a week), and physical activity (moderate and vigorous physical activity ≥5 times a week versus other) through a questionnaire. Body mass index (BMI) was calculated from standing height and weight measured by trained data collectors. Chronic diseases including hypertension, diabetes, and CVDs were categorized as present or absent based on self-report at baseline. The use of ototoxic drugs including aspirin and ibuprofen was also reported by the participants.

The social isolation index was constructed with 3 items: (a) household size (1 point for living alone), (b) frequency of visits to or by friends or family (1 point for less than once a month), and (c) engagement in leisure or social activities (1 point for no participation at least weekly). The total score ranged from 0 to 3, and individuals were categorized as “socially isolated” if they scored 2 or more points [36]. Depressed mood in the past 2 weeks was assessed using the Patient Health Questionnaire and classified as low (not at all, several days) and high (more than half of the days, nearly every day) [37].

### Statistical analysis

Descriptive statistics are presented as mean (SD) and number (percentage) for continuous and categorical variables, respectively. We used Cox proportional hazards regression models to evaluate the association between loneliness and incident HL, then reported using hazard ratio (HR) and 95% confidence interval (CI). All models included age and sex as covariates. Model 1 incorporated age and sex only. In addition to these 2 variables, other models included ethnicity, education, household income, and Townsend index (model 2); smoking, drinking, and physical activity (model 3); BMI and chronic diseases (model 4); social isolation (model 5); depressed mood (model 6); ototoxic drug use status (model 7); genetic risk (model 8). Model 9 was fully adjusted by including all the covariates mentioned above. To assess the extent to which the variables in each model accounted for the association between loneliness and risk of incident HL, we calculated the percentage of excess risk mediated (PERM) using the following formula:

$$\text{PERM}=\frac{{\text{HR}}_{\text{model}\;1}-{\text{HR}}_{\text{model}>1}}{{\text{HR}}_{\text{model}\;1}-1}\times 100\%$$ (1)

Several additional analyses were undertaken to further elucidate the associations between loneliness and risk of incident HL. First, we investigated the associations between loneliness and the risk of different subtypes of HL, including sensorineural, conductive, and mixed HL. Second, we explored whether sex, chronic disease status, social isolation, or genetic risk moderated the relationship between loneliness and HL. This was examined by incorporating interaction terms between loneliness and these variables in the fully adjusted model. Upon observing significant interactions, stratified analyses were conducted based on the levels of these variables. Third, a joint analysis was further performed to directly compare groups with different combinations of genetic risk and loneliness levels.

Several sensitivity analyses were conducted to test the robustness of the findings. First, we used multivariate imputation by chained equations (MICE) in R to impute missing exposure data and then repeated the analyses [38]. Second, to minimize the potential influence of reverse causality, we performed time-lag analyses by excluding cases of HL occurring within the initial 2 years of enrollment. Third, the Fine–Gray subdistribution hazard model was used to take the competing risk of death into account [39]. Fourth, we repeated the analyses using self-reported measures to assess HL. Fifth, depressive mood, social isolation, and genetic risk were treated as the continuous variables [40]. Sixth, neuroticism was included as an additional covariate in the fully adjusted model. Detailed information on the self-reported HL and neuroticism is provided in Supplementary Methods. Finally, we performed a propensity score matching analysis without replacement using the MatchIt package in R. A 1:1 nearest-neighbor matching was applied, with a caliper width set at 0.2 [41,42]. Lonely participants were matched to non-lonely counterparts based on covariates included in the fully adjusted model. Balance between groups was assessed using standardized mean difference, with values below 0.10 indicating acceptable covariate balance. All statistical tests were 2-sided, with a significance level of *P* < 0.05 indicating statistical significance. Statistical analyses were performed using the R software (version 4.3.1).

## Results

Among 502,389 participants, we excluded 372 individuals due to inconsistencies between their genetic and self-reported sex. Additionally, individuals with missing data on loneliness (9,857), a diagnosis of HL (1,208), and those who reported complete deafness (87) at or before baseline were excluded (Fig. S1). Consequently, a total of 490,865 individuals were included in the present analyses (Table). The age of participants at baseline ranged from 38 to 73 years, with a mean (SD) age of 56.5 (8.1) years. Of these, 267,075 (54.4%) were female and 463,613 (94.4%) were white. In total, 90,893 (18.5%) participants were classified as being lonely. Lonely individuals, compared with non-lonely counterparts, were slightly younger and more likely to be female. They also exhibited fewer resources, such as lower levels of education and income, higher deprivation, and increased social isolation. Moreover, lonely individuals had a higher prevalence of chronic diseases, engaged in more unhealthy lifestyle behaviors including smoking and physical inactivity, and reported more depressed moods than non-lonely individuals.

**Table.** Baseline characteristics of participants: overall and stratified by loneliness status and incident HL.

	Overall	Loneliness			Incident HL		
		No	Yes	*P*	No	Yes	*P*
	*N* = 490,865	*N* = 399,972	*N* = 90,893		*N* = 479,269	*N* = 11,596	
Follow-up, years							
Mean (SD)	11.7 (2.2)	11.7 (2.2)	11.7 (2.3)	0.196	11.8 (2.1)	8.30 (3.3)	<0.001
Median (IQR)	12.3 (11.3–13.1)	12.3 (11.3–13.1)	12.3 (11.3–13.1)		12.3 (11.4–13.1)	9.0 (6.0–10.9)	
Reported loneliness	90,893 (18.5)	0	90,893 (100)	<0.001	88,466 (18.5)	2,427 (20.9)	<0.001
Incident HL	11,596 (2.4)	9,169 (2.3)	2,427 (2.8)		0	11,596 (100)	
Age, years							
Mean (SD)	56.5 (8.1)	56.8 (8.1)	55.5 (8.1)	<0.001	56.4 (8.1)	61.5 (6.6)	<0.001
Median (IQR)	58.0 (50.0–63.0)	58.0 (50.0–63.0)	56.0 (49.0–62.0)		58.0 (50.0–63.0)	63.0 (58.0–66.0)	
Age groups, years							
0–49	114,734 (23.4)	90,274 (22.6)	24,460 (26.9)	<0.001	113,892 (23.8)	842 (7.3)	<0.001
50–59	163,239 (33.3)	130,700 (32.7)	32,539 (35.8)		160,721 (33.5)	2,518 (21.7)	
60 or older	212,892 (43.4)	178,998 (44.8)	33,894 (37.3)		204,656 (42.7)	8,236 (71.0)	
Sex							
Female	267,075 (54.4)	210,318 (52.6)	56,757 (62.4)	<0.001	261,791 (54.6)	5,284 (45.6)	<0.001
Male	223,790 (45.6)	189,654 (47.4)	34,136 (37.6)		217,478 (45.4)	6,312 (54.4)	
Ethnicity							
White	463,613 (94.4)	379,863 (95.0)	83,750 (92.1)	<0.001	452,489 (94.4)	11,124 (95.9)	<0.001
Other	25,641 (5.2)	18,860 (4.7)	6,781 (7.5)		25,213 (5.3)	428 (3.7)	
Missing	1,611 (0.3)	1,249 (0.3)	362 (0.4)		1,567 (0.3)	44 (0.4)	
Education							
No secondary education	164,428 (33.5)	128,659 (32.2)	35,769 (39.4)	<0.001	159,535 (33.3)	4,893 (42.2)	<0.001
Secondary education	159,449 (32.5)	130,764 (32.7)	28,685 (31.6)		155,877 (32.5)	3,572 (30.8)	
University degree	158,386 (32.3)	133,777 (33.4)	24,609 (27.1)		155,588 (32.5)	2,798 (24.1)	
Missing	8,602 (1.8)	6,772 (1.7)	1,830 (2.0)		8,269 (1.7)	333 (2.9)	
Income levels							
Less than £31,000	205,222 (41.8)	157,646 (39.4)	47,576 (52.3)	<0.001	198,917 (41.5)	6,305 (54.4)	<0.001
At least £31,000	223,607 (45.6)	192,585 (48.1)	31,022 (34.1)		220,169 (45.9)	3,438 (29.6)	
Missing	62,036 (12.6)	49,741 (12.4)	12,295 (13.5)		60,183 (12.6)	1,853 (16.0)	
Townsend deprivation index							
Mean (SD)	−1.3 (3.1)	−1.5 (3.0)	−0.5 (3.4)	<0.001	−1.3 (3.1)	−1.2 (3.2)	<0.001
Missing	606 (0.1)	447 (0.1)	159 (0.2)		593 (0.1)	13 (0.1)	
BMI, kg/m^2^
Mean (SD)	27.4 (4.8)	27.3 (4.6)	28.1 (5.4)	<0.001	27.4 (4.8)	28.1 (4.8)	<0.001
Missing	2,445 (0.5)	1,764 (0.4)	681 (0.7)		2,368 (0.5)	77 (0.7)	
Smoking status							
Never	267,871 (54.6)	221,288 (55.3)	46,583 (51.3)	<0.001	262,465 (54.8)	5,406 (46.6)	<0.001
Past	169,928 (34.6)	139,969 (35.0)	29,959 (33.0)		164,993 (34.4)	4,935 (42.6)	
Current	51,555 (10.5)	37,555 (9.4)	14,000 (15.4)		50,370 (10.5)	1,185 (10.2)	
Missing	1,511 (0.3)	1,160 (0.3)	351 (0.4)		1,441 (0.3)	70 (0.6)	
Alcohol intake							
Twice or less per week	276,740 (56.4)	225,711 (56.4)	51,029 (56.1)	<0.001	270,219 (56.4)	6,521 (56.2)	0.062
At least 3 times per week	213,742 (43.5)	174,018 (43.5)	39,724 (43.7)		208,683 (43.5)	5,059 (43.6)	
Missing	383 (0.1)	243 (0.1)	140 (0.2)		367 (0.1)	16 (0.1)	
Physical activity							
Low	269,267 (54.9)	219,796 (55.0)	49,471 (54.4)	<0.001	263,366 (55.0)	5,901 (50.9)	<0.001
High	191,325 (39.0)	157,498 (39.4)	33,827 (37.2)		186,615 (38.9)	4,710 (40.6)	
Missing	30,273 (6.2)	22,678 (5.7)	7,595 (8.4)		29,288 (6.1)	985 (8.5)	
Hypertension							
No	348,474 (71.0)	286,180 (71.6)	62,294 (68.5)	<0.001	341,598 (71.3)	6,876 (59.3)	<0.001
Yes	142,177 (29.0)	113,660 (28.4)	28,517 (31.4)		137,467 (28.7)	4,710 (40.6)	
Missing	214 (0.0)	132 (0.0)	82 (0.1)		204 (0.0)	10 (0.1)	
Diabetes							
No	464,705 (94.7)	380,305 (95.1)	84,400 (92.9)	<0.001	454,260 (94.8)	10,445 (90.1)	<0.001
Yes	25,939 (5.3)	19,531 (4.9)	6,408 (7.1)		24,797 (5.2)	1,142 (9.8)	
Missing	221 (0.0)	136 (0.0)	85 (0.1)		212 (0.0)	9 (0.1)	
Cardiovascular disease							
No	461,563 (94.0)	377,653 (94.4)	83,910 (92.3)	<0.001	451,375 (94.2)	10,188 (87.9)	<0.001
Yes	28,331 (5.8)	21,638 (5.4)	6,693 (7.4)		26,957 (5.6)	1,374 (11.8)	
Missing	971 (0.2)	681 (0.2)	290 (0.3)		937 (0.2)	34 (0.3)	
Socially isolated							
No	436,325 (88.9)	363,336 (90.8)	72,989 (80.3)	<0.001	426,245 (88.9)	10,080 (86.9)	<0.001
Yes	43,829 (8.9)	28,668 (7.2)	15,161 (16.7)		42,721 (8.9)	1,108 (9.6)	
Missing	10,711 (2.2)	7,968 (2.0)	2,743 (3.0)		10,303 (2.1)	408 (3.5)	
Depressed mood							
Low	446,605 (91.0)	376,549 (94.1)	70,056 (77.1)	<0.001	436,248 (91.0)	10,357 (89.3)	<0.001
High	23,480 (4.8)	8,785 (2.2)	14,695 (16.2)		22,816 (4.8)	664 (5.7)	
Missing	20,780 (4.2)	14,638 (3.7)	6,142 (6.8)		20,205 (4.2)	575 (5.0)	
Use of ototoxic drugs							
No	350,390 (71.4)	289,390 (72.4)	61,000 (67.1)	<0.001	342,895 (71.5)	7,495 (64.6)	<0.001
Yes	131,459 (26.8)	103,538 (25.9)	27,921 (30.7)		127,695 (26.6)	3,764 (32.5)	
Missing	9,016 (1.8)	7,044 (1.8)	1,972 (2.2)		8,679 (1.8)	337 (2.9)	
Genetic risk							
Low	95,644 (19.5)	77,637 (19.4)	18,007 (19.8)	<0.001	93,819 (19.6)	1,825 (15.7)	<0.001
Intermediate	288,031 (58.7)	235,134 (58.8)	52,897 (58.2)		281,278 (58.7)	6,753 (58.2)	
High	92,760 (18.9)	75,752 (18.9)	17,008 (18.7)		90,128 (18.8)	2,632 (22.7)	
Missing	14,430 (2.9)	11,449 (2.9)	2,981 (3.3)		14,044 (2.9)	386 (3.3)	

BMI, body mass index; HL, hearing loss; IQR, interquartile range

**Fig. 1.**
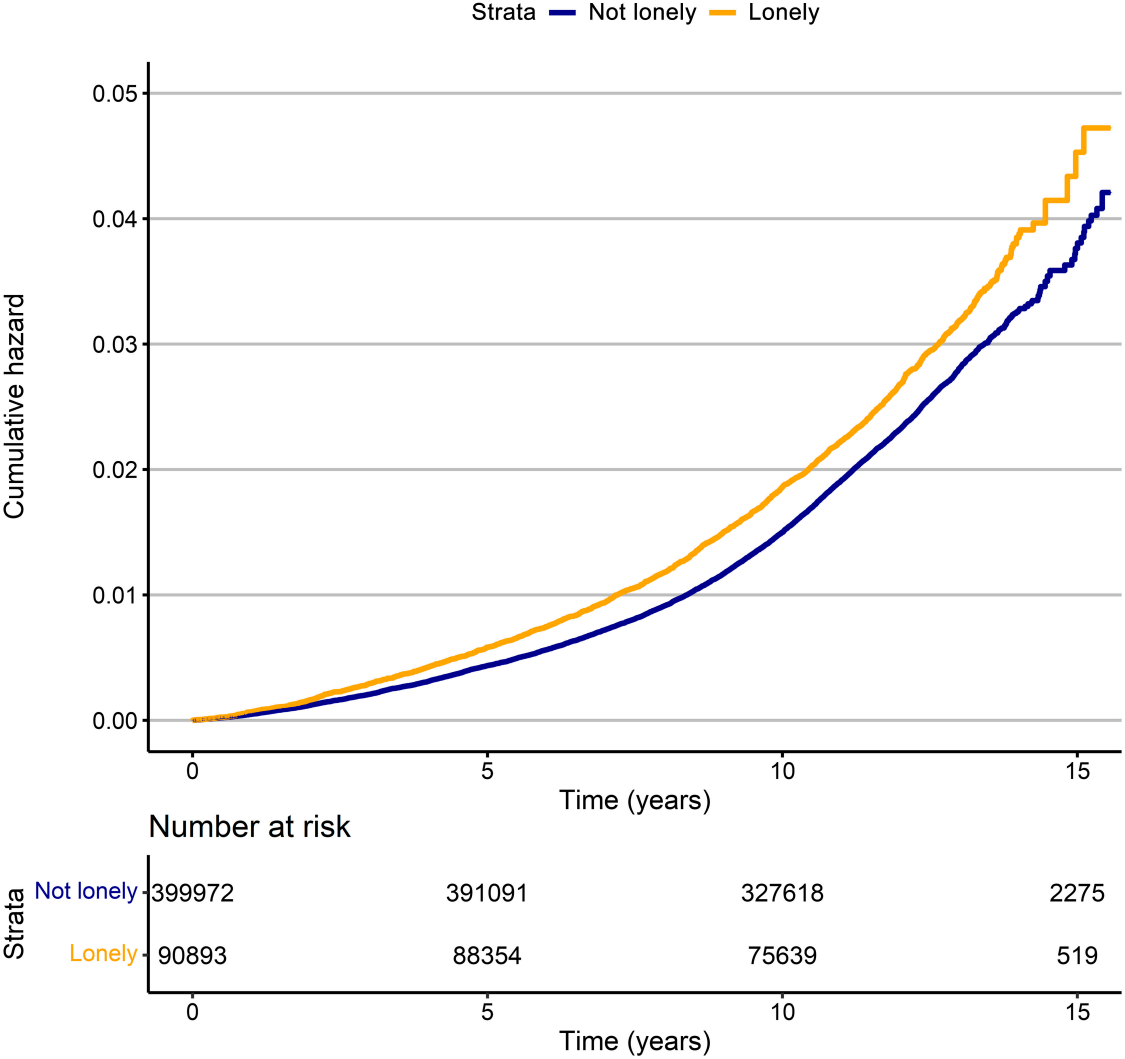
Cumulative hazard of HL among participants who did and did not report loneliness at baseline.

**Fig. 2.**
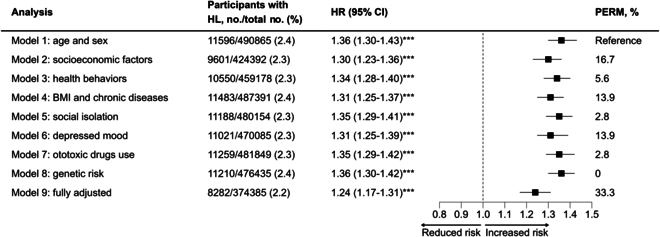
Association of loneliness with incident HL in models with different sets of covariates and the fully adjusted model. ***P < 0.001. Notes: The numbers of total participants and participants with HL vary across models because of missing data for the covariates. Age and sex were included as covariates in all models.

During a total of 5,732,222 person-years of follow-up [a median (interquartile range) length of 12.3 (11.3 to 13.1) years], 11,596 participants (2.4%) developed HL. Figure 1 presents the cumulative hazard of HL among participants who did and did not report loneliness. As shown in Fig. 2, lonely participants, in comparison with non-lonely ones, had a higher risk of HL after adjusting for age and sex (model 1: HR, 1.36; 95% CI, 1.30 to 1.43), and this association was significant across all models. After adjusting for all covariates, the HR attenuated to 1.24 (95% CI, 1.17 to 1.31) but remained significant (Fig. 2). Model 3 (health behaviors), 5 (social isolation), 7 (ototoxic drug use status), and 8 (genetic risk of HL) showed no substantial attenuation of the association between loneliness and HL risk compared to model 1 (age and sex). However, notable attenuation was observed after adjusting for chronic diseases and BMI (model 4: PERM, 13.9%), depressed mood (model 6: PERM, 13.9%), and socioeconomic factors (model 2: PERM, 16.7%), respectively.

During the follow-up period, 357 individuals developed conductive HL, 1,294 developed sensorineural HL, and 188 developed mixed HL. After controlling for all covariates, no significant associations were found between loneliness and the risk of conductive or mixed HL (both *P*s > 0.05). However, loneliness was significantly associated with an increased risk of incident sensorineural HL (HR, 1.23; 95% CI, 1.07 to 1.42) (Table S3).

We found no significant interaction effects between loneliness and chronic diseases (*P*_interaction_ = 0.773), social isolation (*P*_interaction_ = 0.787), or genetic risk (*P*_interaction_ = 0.831). However, sex (*P*_interaction_ = 0.006) emerged as a significant moderator of the association between loneliness and incident HL. Women (HR, 1.30; 95% CI, 1.20 to 1.41) exhibited a stronger association compared to men (HR, 1.18; 95% CI, 1.09 to 1.29) (Fig. S3).

**Fig. 3.**
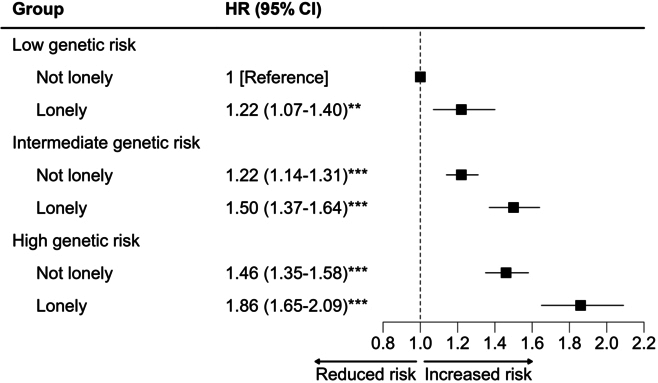
Joint association of loneliness and genetic risk with incident HL. **P < 0.01, ***P < 0.001 Notes: Analysis was adjusted for age, sex, ethnicity, education, household income, Townsend deprivation index, smoking status, alcohol intake status, physical activity, hypertension, diabetes, cardiovascular disease, social isolation, depressed mood, and use of ototoxic drugs.

The risk of incident HL increased monotonically across genetic risk quintiles (Table S4). When genetic risk and loneliness categories were combined, there was also a monotonic association of increasing genetic risk and loneliness with higher HL risk. Across genetic risk groups, loneliness was associated with elevated absolute risks of incident events. The highest risk of incident HL was observed among individuals with high genetic risk of HL and loneliness (HR, 1.86; 95% CI, 1.65 to 2.09) (Fig. 3).

The associations between loneliness and incident HL remained robust across several sensitivity analyses using fully adjusted models. These encompassed analyses based on imputed data (HR, 1.19; 95% CI, 1.13 to 1.25), a time-lag analysis excluding HL cases within the initial 2 years of follow-up (HR, 1.24; 95% CI, 1.17 to 1.32), and a Fine–Gray model accounting for the competing risk of death (HR, 1.24; 95% CI, 1.17 to 1.31). Additional analyses using self-reported measurements to assess HL (HR, 1.14; 95% CI, 1.07 to 1.21), treating depression, social isolation, and genetic risk as continuous variables (HR, 1.18; 95% CI, 1.11 to 1.26), and adjusting for neuroticism (HR, 1.15; 95% CI, 1.08 to 1.24) further supported the robustness of the findings (Tables S5 to S10). Furthermore, following propensity score matching, the lonely and non-lonely groups were well-balanced across all covariates, with standardized mean differences for all variables remaining below 0.10 (Table S11). The association between loneliness and an increased risk of HL persisted in the matched cohorts (HR, 1.23; 95% CI, 1.15 to 1.33) (Table S12).

## Discussion

In this large community-based study involving over 490,000 individuals, loneliness was associated with a 24% increased risk of new-onset HL, even after accounting for multiple confounding factors, including demographic, biological, and lifestyle factors, social isolation, depression, chronic diseases, use of ototoxic medication, and genetic risk for HL. When examining HL subtypes, loneliness was specifically linked to a higher risk of sensorineural HL. Furthermore, the relationship was moderated by sex, with stratified analysis indicating a stronger association among women than men. In contrast, genetic risk for HL did not substantially influence the association between loneliness and HL.

To our knowledge, this is the first study to examine the association between loneliness and the risk of incident HL. Although previous studies have explored the relationship between these 2 variables, they primarily focus on how HL contributes to heightened loneliness. For instance, a small-scale study found a cross-sectional association between HL and elevated loneliness [43], while a larger longitudinal study based on the English Longitudinal Study of Ageing (ELSA) reported similar findings [44]. The notion that individuals with HL are prone to experiencing loneliness is further supported by 2 recent reviews [21,22]. In contrast, our findings introduce a novel perspective: Loneliness could also lead to an increased risk of HL. This suggests a bidirectional association, implying a potentially vicious cycle between the experience of loneliness and hearing impairment. Although increased social isolation has also been observed among individuals with HL [21,22], our study did not find evidence that social isolation influenced HL risk after accounting for potential confounders (Table S2). This suggests that subjective feelings of loneliness, rather than objective deficiencies in social relationships, may play a more important role in influencing auditory health.

We found a monotonic increase in the risk of HL with rising levels of genetic risk and loneliness. While loneliness did not exacerbate the impact of genetic risk on HL, individuals with both high genetic risk and loneliness exhibited the highest incidence of HL. Given the heightened susceptibility in this subgroup, future intervention policies targeting loneliness alleviation may need to prioritize these individuals.

This study tested a broad range of traditional HL risk factors that could potentially mediate the impact of loneliness. While lonely individuals were more likely to engage in unhealthy behaviors such as physical inactivity and smoking [26], our analysis did not indicate a notable mediating effect of these factors. Adjustments for BMI and chronic diseases, as well as depressed mood, each led to a 13.9% attenuation in the relationship, implying that physical and mental health are equally potent mediators. Socioeconomic status emerged as the strongest mediator, which resulted in a 16.7% reduction in the association. This aligns with findings from a previous UK Biobank study, which found that the associations of loneliness with CVD and mortality were largely explained by socioeconomic status [45]. Accumulated evidence suggests that loneliness can lead to socioeconomic adversity [46,47], which in turn contributes to adverse health outcomes. In contrast, other factors such as social isolation, use of ototoxic drugs, and genetic risk of HL did not markedly diminish the relationship between loneliness and HL.

When all covariates were considered, the relationship between loneliness and HL was attenuated by 33.3%. This suggests that, beyond the identified mediators, other physiological pathways may contribute to the excess risk. For example, loneliness has been related to maladaptive stress responses including increased activity in the hypothalamic–pituitary–adrenal axis and the sympathetic nervous system [28]. These exaggerated stress reactivities may impair both central and peripheral auditory systems [48,49]. Loneliness has also been associated with increased oxidative stress [50], which could contribute to hearing impairment [51]. Furthermore, it has been linked to elevated inflammation and blood pressure, both of which could further explain the heightened HL risk [24,27,49,52].

Subtype analyses revealed a significant association between loneliness and sensorineural HL, but not conductive HL, which reinforced the plausibility of these mechanisms. Conductive HL predominantly arises from mechanical disruptions in sound transmission, such as cerumen impaction, otitis media, or structural pathologies like otosclerosis-induced stapes fixation [10]. These conditions are primarily influenced by environmental or anatomical factors, offering limited pathways for psychosocial effects. In contrast, sensorineural HL involves damage to the sensory or neural components of the peripheral auditory system, which may be more vulnerable to the neuroendocrine, inflammatory, or behavioral changes associated with loneliness [24,27,28,53].

Our study found a stronger association between loneliness and risk of HL in women compared to men. This finding is consistent with a previous study linking psychological distress to hearing problems [54]. Similarly, several studies have indicated that lonely women experience higher rates of CVD and diabetes than men [55,56]. This suggests that women may be more vulnerable to the health impacts of loneliness, potentially leading to a higher risk of HL. This hypothesis is also supported by evidence suggesting that lonely women exhibit more pronounced neuroendocrine, cardiovascular, and inflammatory stress responses than lonely men [57,58].

The strengths of our study included its large sample size, long-term follow-up, comprehensive adjustment for covariates, and independent verification of HL diagnoses using health records. However, several limitations must be acknowledged. First, loneliness was assessed using a single question with a dichotomous answer. While this single-item measure correlates strongly with validated multi-item scales [31], its binary nature may limit sensitivity, potentially overlooking variations in the frequency or intensity of loneliness [59]. Additionally, including the term “lonely” in the question may introduce contextual biases based on respondents’ interpretations [60]. Future studies should consider using multi-item scales, such as the UCLA loneliness scale, to enhance measurement accuracy. Second, our reliance on hospital admission and death records to identify HL cases may have led to underestimation, as individuals with early-stage HL could have been missed. Although we validated findings using self-reported HL, future research should incorporate more objective diagnostic methods, such as pure-tone audiometry and otoacoustic emissions, to enhance case ascertainment. Third, since most UK Biobank participants were of European descent, caution is warranted when generalizing these findings to other ethnic groups. Finally, as an observational study, causal inferences cannot be established.

## Conclusion

In conclusion, this study highlights a notable association between loneliness and an increased risk of HL, particularly sensorineural HL. These findings underscore the potential bidirectional relationship between loneliness and HL and suggest that addressing loneliness could be an important strategy in HL prevention efforts. Future research should further explore underlying mechanisms and evaluate interventions aimed at reducing loneliness to mitigate HL risk.

## Ethical Approval

The UK Biobank was approved by the National Health Service National Research Ethics Service (21/NW/0157). At recruitment, all participants gave informed consent to participate and be followed-up through data-linkage.

## Data Availability

Data are available in a public, open access repository. The UK Biobank data are available on application to the UK Biobank (www.ukbiobank.ac.uk/).

## References

[1] Manchaiah VKC, Stephens D. Perspectives on defining ‘hearing loss’ and its consequences. Hear Balance Commun. 2013;11(1):6–16.

[2] World Health Organization. *World report on hearing*. Geneva: World Health Organization; 2021.

[3] World Health Organization. *Global costs of unaddressed hearing loss and cost-effectiveness of interventions: A WHO report, 2017*. Geneva: World Health Organization; 2017.

[4] Haile LM, Kamenov K, Briant PS, Orji AU, Steinmetz JD, Abdoli A, Abdollahi M, Abu-Gharbieh E, Afshin A, Ahmed H, et al. Hearing loss prevalence and years lived with disability, 1990–2019: Findings from the Global Burden of Disease Study 2019. Lancet. 2021;397(10278):996–1009. 33714390 10.1016/S0140-6736(21)00516-X PMC7960691

[5] Lawrence BJ, Jayakody DMP, Bennett RJ, Eikelboom RH, Gasson N, Friedland PL. Hearing loss and depression in older adults: A systematic review and meta-analysis. Gerontologist. 2020;60(3):e137–e154. 30835787 10.1093/geront/gnz009

[6] Lin FR, Yaffe K, Xia J, Xue QL, Harris TB, Purchase-Helzner E, Satterfield S, Ayonayon HN, Ferrucci L, Simonsick EM, et al. Hearing loss and cognitive decline in older adults. JAMA Intern Med. 2013;173(4):293–299. 23337978 10.1001/jamainternmed.2013.1868 PMC3869227

[7] Yévenes-Briones H, Caballero FF, Struijk EA, Rey-Martinez J, Montes-Jovellar L, Graciani A, Rodríguez-Artalejo F, Lopez-Garcia E. Association between hearing loss and impaired physical function, frailty, and disability in older adults: A cross-sectional study. JAMA Otolaryngol Head Neck Surg. 2021;147(11):951–958. 34554203 10.1001/jamaoto.2021.2399 PMC8461549

[8] Tan CJ-W, Koh JWT, Tan BKJ, Woon CY, Teo YH, Ng LS, Loh WS. Association between hearing loss and cardiovascular disease: A meta-analysis. Otolaryngol Head Neck Surg. 2023;179(3):694–707. 10.1002/ohn.599 38063267

[9] Livingston G, Huntley J, Sommerlad A, Ames D, Ballard C, Banerjee S, Brayne C, Burns A, Cohen-Mansfield J, Cooper C, et al. Dementia prevention, intervention, and care: 2020 report of the lancet commission. Lancet. 2020;396(10248):413–446. 32738937 10.1016/S0140-6736(20)30367-6 PMC7392084

[10] Cunningham LL, Tucci DL. Hearing loss in adults. N Engl J Med. 2017;377(25):2465–2473. 29262274 10.1056/NEJMra1616601 PMC6457651

[11] Dawes P, Cruickshanks KJ, Moore DR, Edmondson-Jones M, McCormack A, Fortnum H, Munro KJ. Cigarette smoking, passive smoking, alcohol consumption, and hearing loss. J Assoc Res Otolaryngol. 2014;15(4):663–674. 24899378 10.1007/s10162-014-0461-0 PMC4141428

[12] Lin BM, Curhan SG, Wang M, Eavey R, Stankovic KM, Curhan GC. Hypertension, diuretic use, and risk of hearing loss. Am J Med. 2016;129(4):416–422. 26656761 10.1016/j.amjmed.2015.11.014 PMC4792671

[13] Samocha-Bonet D, Wu B, Ryugo DK. Diabetes mellitus and hearing loss: A review. Ageing Res Rev. 2021;71: Article 101423. 34384902 10.1016/j.arr.2021.101423

[14] Yévenes-Briones H, Caballero FF, Banegas JR, Rodríguez-Artalejo F, Lopez-Garcia E. Association of lifestyle behaviors with hearing loss: The UK Biobank cohort study. Mayo Clin Proc. 2022;97(11):2040–2049. 35710463 10.1016/j.mayocp.2022.03.029

[15] Curhan SG, Eavey R, Wang M, Stampfer MJ, Curhan GC. Body mass index, waist circumference, physical activity, and risk of hearing loss in women. Am J Med. 2013;126(12):1142.e1–1142.e8. 10.1016/j.amjmed.2013.04.026 PMC3848606 24125639

[16] Peplau LA, Perlman D. Perspectives on loneliness. In: *Loneliness: A sourcebook of current theory, research and therapy*. New York (NY): Wiley; 1982. p. 1–18.

[17] Holt-Lunstad J, Smith TB, Baker M, Harris T, Stephenson D. Loneliness and social isolation as risk factors for mortality: A meta-analytic review. Perspect Psychol Sci. 2015;10(2):227–237. 25910392 10.1177/1745691614568352

[18] Academies National of Sciences, Engineering, and Medicine, *Social isolation and loneliness in older adults: Opportunities for the health care system*. Washington (DC): The National Academies Press; 2020. 32510896

[19] World Health Organization. WHO launches commission to foster social connection. 2023. https://www.who.int/news/item/15-11-2023-who-launches-commission-to-foster-social-connection.

[20] Valtorta NK, Kanaan M, Gilbody S, Ronzi S, Hanratty B. Loneliness and social isolation as risk factors for coronary heart disease and stroke: Systematic review and meta-analysis of longitudinal observational studies. Heart. 2016;102(13):1009–1016. 27091846 10.1136/heartjnl-2015-308790 PMC4941172

[21] Shukla A, Harper M, Pedersen E, Goman A, Suen JJ, Price C, Applebaum J, Hoyer M, Lin FR, Reed NS. Hearing loss, loneliness, and social isolation: A systematic review. Otolaryngol Head Neck Surg. 2020;162(5):622–633. 32151193 10.1177/0194599820910377 PMC8292986

[22] Bott A, Saunders G. A scoping review of studies investigating hearing loss, social isolation and/or loneliness in adults. Int J Audiol. 2021;60(sup2):30–46. 34030565 10.1080/14992027.2021.1915506

[23] Hackett RA, Hudson JL, Chilcot J. Loneliness and type 2 diabetes incidence: Findings from the English Longitudinal Study of Ageing. Diabetologia. 2020;63(11):2329–2338. 32929525 10.1007/s00125-020-05258-6 PMC7527325

[24] Hawkley LC, Thisted RA, Masi CM, Cacioppo JT. Loneliness predicts increased blood pressure: 5-year cross-lagged analyses in middle-aged and older adults. Psychol Aging. 2010;25(1):132–141. 20230134 10.1037/a0017805 PMC2841310

[25] Erzen E, Çikrikci Ö. The effect of loneliness on depression: A meta-analysis. Int J Soc Psychiatry. 2018;64(5):427–435. 29792097 10.1177/0020764018776349

[26] Shankar A, McMunn A, Banks J, Steptoe A. Loneliness, social isolation, and behavioral and biological health indicators in older adults. Health Psychol. 2011;30(4):377–385. 21534675 10.1037/a0022826

[27] Miyata J, Umesawa M, Yoshioka T, Iso H. Association between high systolic blood pressure and objective hearing impairment among Japanese adults: A facility-based retrospective cohort study. Hypertens Res. 2022;45(1):155–161. 34690351 10.1038/s41440-021-00737-8

[28] Cacioppo JT, Cacioppo S, Capitanio JP, Cole SW. The neuroendocrinology of social isolation. Annu Rev Psychol. 2015;66(1):733–767. 25148851 10.1146/annurev-psych-010814-015240 PMC5130104

[29] Trpchevska N, Freidin MB, Broer L, Oosterloo BC, Yao S, Zhou Y, Vona B, Bishop C, Bizaki-Vallaskangas A, Canlon B, et al. Genome-wide association meta-analysis identifies 48 risk variants and highlights the role of the stria vascularis in hearing loss. Am J Hum Genet. 2022;109(6):1077–1091. 35580588 10.1016/j.ajhg.2022.04.010 PMC9247887

[30] Sudlow C, Gallacher J, Allen N, Beral V, Burton P, Danesh J, Downey P, Elliott P, Green J, Landray M, et al. UK biobank: An open access resource for identifying the causes of a wide range of complex diseases of middle and old age. PLOS Med. 2015;12(3): Article e1001779. 25826379 10.1371/journal.pmed.1001779 PMC4380465

[31] Hughes ME, Waite LJ, Hawkley LC, Cacioppo JT. A short scale for measuring loneliness in large surveys: Results from two population-based studies. Res Aging. 2004;26(6):655–672. 18504506 10.1177/0164027504268574 PMC2394670

[32] Osler M, Christensen GT, Mortensen EL, Christensen K, Garde E, Rozing MP. Hearing loss, cognitive ability, and dementia in men age 19–78 years. Eur J Epidemiol. 2019;34(2):125–130. 30306425 10.1007/s10654-018-0452-2

[33] Clifford RE, Maihofer AX, Chatzinakos C, Coleman JRI, Daskalakis NP, Gasperi M, Hogan K, Mikita EA, Stein MB, Tcheandjieu C, et al. Genetic architecture distinguishes tinnitus from hearing loss. Nat Commun. 2024;15(1):614. 38242899 10.1038/s41467-024-44842-x PMC10799010

[34] Lourida I, Hannon E, Littlejohns TJ, Langa KM, Hyppönen E, Kuzma E, Llewellyn DJ. Association of lifestyle and genetic risk with Incidence of dementia. JAMA. 2019;322(5):430–437. 31302669 10.1001/jama.2019.9879 PMC6628594

[35] Gilthorpe MS. The importance of normalisation in the construction of deprivation indices. J Epidemiol Community Health. 1995;49(Suppl 2):S45–S50. 8594134 10.1136/jech.49.suppl_2.s45 PMC1060876

[36] Terracciano A, Luchetti M, Karakose S, Stephan Y, Sutin AR. Loneliness and risk of Parkinson disease. JAMA Neurol. 2023;80(11):1138–1144. 37782489 10.1001/jamaneurol.2023.3382 PMC10546293

[37] Elovainio M, Komulainen K, Sipilä PN, Pulkki-Råback L, Cachón Alonso L, Pentti J, Nyberg ST, Suominen S, Vahtera J, Lipsanen J, et al. Association of social isolation and loneliness with risk of incident hospital-treated infections: An analysis of data from the UK Biobank and Finnish Health and Social Support studies. Lancet Public Health. 2023;8(2):e109–e118. 36669514 10.1016/S2468-2667(22)00253-5 PMC9879771

[38] Liang YY, Chen Y, Feng H, Liu X, Ai QYH, Xue H, Shu X, Weng F, He Z, Ma J, et al. Association of social isolation and loneliness with incident heart failure in a population-based cohort study. Heart Fail. 2023. 11(3):334–344. 10.1016/j.jchf.2022.11.028 PMC9891238 36737310

[39] Wang X, Ma H, Li X, Heianza Y, Fonseca V, Qi L. Joint association of loneliness and traditional risk factor control and incident cardiovascular disease in diabetes patients. Eur Heart J. 2023;44(28):2583–2591. 37385629 10.1093/eurheartj/ehad306 PMC10361009

[40] Sarris J, Thomson R, Hargraves F, Eaton M, de Manincor M, Veronese N, Solmi M, Stubbs B, Yung AR, Firth J. Multiple lifestyle factors and depressed mood: A cross-sectional and longitudinal analysis of the UK Biobank (N = 84,860). BMC Med. 2020;18(1):354. 33176802 10.1186/s12916-020-01813-5 PMC7661271

[41] Austin PC, Stuart EA. The performance of inverse probability of treatment weighting and full matching on the propensity score in the presence of model misspecification when estimating the effect of treatment on survival outcomes. Stat Methods Med Res. 2017;26(4):1654–1670. 25934643 10.1177/0962280215584401 PMC5564952

[42] Xie J, Feng S, Li X, Gea-Mallorquí E, Prats-Uribe A, Prieto-Alhambra D. Comparative effectiveness of the BNT162b2 and ChAdOx1 vaccines against Covid-19 in people over 50. Nat Commun. 2022;13(1):1519. 35314696 10.1038/s41467-022-29159-x PMC8938429

[43] Sung Y-K et al. Association of hearing loss and loneliness in older adults. J Aging Health. 2015;28(6):979–994. 26597841 10.1177/0898264315614570

[44] Maharani A, Pendleton N, Leroi I. Hearing impairment, loneliness, social isolation, and cognitive function: Longitudinal analysis using English longitudinal study on ageing. Am J Geriatr Psychiatry. 2019;27(12):1348–1356. 31402088 10.1016/j.jagp.2019.07.010

[45] Christian H, Pulkki-Rback L, Virtanen M, Jokela M, Kivimaki M, Elovainio M. Social isolation and loneliness as risk factors for myocardial infarction, stroke and mortality: UK Biobank cohort study of 479 054 men and women. Heart. 2018;104(18):1536. 29588329 10.1136/heartjnl-2017-312663

[46] Morrish N, Medina-Lara A. Does unemployment lead to greater levels of loneliness? A systematic review. Soc Sci Med. 2021;287: Article 114339. 34455335 10.1016/j.socscimed.2021.114339 PMC8505794

[47] Bryan BT, Thompson KN, Goldman-Mellor S, Moffitt TE, Odgers CL, So SLS, Uddin Rahman M, Wertz J, Matthews T, Arseneault L. The socioeconomic consequences of loneliness: Evidence from a nationally representative longitudinal study of young adults. Soc Sci Med. 2024;345: Article 116697. 38490911 10.1016/j.socscimed.2024.116697 PMC11845567

[48] Tian C, Zha D. Sympathetic nervous system regulation of auditory function. Audiol Neurotol. 2021;27(2):93–103. 10.1159/000517452 34407531

[49] Nash SD, Cruickshanks KJ, Zhan W, Tsai MY, Klein R, Chappell R, Nieto FJ, Klein BEK, Schubert CR, Dalton DS, et al. Long-term assessment of systemic inflammation and the cumulative incidence of age-related hearing impairment in the epidemiology of hearing loss study. J Gerontol A Biol Sci Med Sci. 2014;69A(2):207–214. 10.1093/gerona/glt075 PMC4038239 23739996

[50] Li H, Xia N. The role of oxidative stress in cardiovascular disease caused by social isolation and loneliness. Redox Biol. 2020;37: Article 101585. 32709420 10.1016/j.redox.2020.101585 PMC7767744

[51] Henderson D, Bielefeld EC, Harris KC, Hu BH. The role of oxidative stress in noise-induced hearing loss. Ear Hear. 2006;27(1):1–19. 16446561 10.1097/01.aud.0000191942.36672.f3

[52] Nersesian PV, Han HR, Yenokyan G, Blumenthal RS, Nolan MT, Hladek MD, Szanton SL. Loneliness in middle age and biomarkers of systemic inflammation: Findings from Midlife in the United States. Soc Sci Med. 2018;209:174–181. 29735350 10.1016/j.socscimed.2018.04.007 PMC6013269

[53] Schreiber BE, Agrup C, Haskard DO, Luxon LM. Sudden sensorineural hearing loss. Lancet. 2010;375(9721):1203–1211. 20362815 10.1016/S0140-6736(09)62071-7

[54] Herr RM, Bosch JA, Theorell T, Loerbroks A. Bidirectional associations between psychological distress and hearing problems: An 18-year longitudinal analysis of the British Household Panel Survey. Int J Audiol. 2018;57(11):816–824. 30052099 10.1080/14992027.2018.1490034

[55] Thurston RC, Kubzansky LD. Wowen, Loneliness, and incident coronary heart disease. Psychosom Med. 2009;71(8):836–842. 19661189 10.1097/PSY.0b013e3181b40efc PMC2851545

[56] Christiansen J, Larsen FB, Lasgaard M. Do stress, health behavior, and sleep mediate the association between loneliness and adverse health conditions among older people? Soc Sci Med. 2016;152:80–86. 26849687 10.1016/j.socscimed.2016.01.020

[57] Steptoe A, Owen N, Kunz-Ebrecht SR, Brydon L. Loneliness and neuroendocrine, cardiovascular, and inflammatory stress responses in middle-aged men and women. Psychoneuroendocrinology. 2004;29(5):593–611. 15041083 10.1016/S0306-4530(03)00086-6

[58] Hackett RA, Hamer M, Endrighi R, Brydon L, Steptoe A. Loneliness and stress-related inflammatory and neuroendocrine responses in older men and women. Psychoneuroendocrinology. 2012;37(11):1801–1809. 22503139 10.1016/j.psyneuen.2012.03.016

[59] Tabachnick BG, Fidell LS, Ullman JB, *Using multivariate statistics (vol. 5)*. Boston (MA): Pearson; 2007.

[60] Routasalo P, Pitkala KH. Loneliness among older people. Rev Clin Gerontol. 2003;13(4):303–311.

