# Supplementary material for: Loneliness and Risk of Incident Hearing Loss: The UK Biobank Study
Source: Health Data Sci. 2025 May 2;5:0281. doi: 10.34133/hds.0281 (PMC12046134; doi:10.34133/hds.0281)
Supplement: Supplementary 1 — Supplementary Methods Tables S1 to S12 Figs. S1 to S3 [file hds.0281.f1.docx]

**Supplementary appendix**

**Title: Loneliness and risk of incident hearing loss: the UK Biobank cohort study**

**Supplementary Methods**

**Table S1.** Single nucleotide polymorphisms (SNPs) used to build the genetic risk score for hearing loss.

**Table S2.** Hazard ratios of incident hearing loss for all predictors in the fully adjusted model.

**Table S3.** Associations of loneliness and risks of various subtypes of hearing loss.

**Table S4.** Risk of incident hearing loss according to genetic risk quintile.

**Table S5.** Association of loneliness and risk of incident hearing loss in the sample with imputed data on exposures.

**Table S6.** Association of loneliness and risk of incident hearing loss after excluding hearing loss events occurring within the first two years.

**Table S7.** Association of loneliness and risk of incident hearing loss from the Fine-Gray subdistribution Hazard Model.

**Table S8.** Association of loneliness and risk of incident self-reported hearing loss.

**Table S9.** Association of loneliness and risk of incident hearing loss after treating depressive mood, social isolation, and genetic risk as continuous variables.

**Table S10.** Association of loneliness and risk of incident hearing loss with neuroticism included as an additional covariate.

**Table S11.** Baseline characteristics of participants after propensity score matching.

**Table S12.** Association of loneliness and risk of incident hearing loss after propensity score matching.

**Figure S1.** Flowchart of participants selection

**Figure S2.** Distribution of the genetic risk score for hearing loss.

**Figure S3.** Associations of loneliness and risk of incident hearing loss across subgroups of sex, chronic diseases, social isolation, and genetic risk.

**Supplementary Methods**

***Detailed information of the analyses based on self-reported hearing loss (HL).***

In the UK Biobank, self-reported HL were assessed by three questions [1]: (1) “Do you use a hearing aid most of the time?”, (2) “Do you have any difficulty with your hearing?”, and (3) “Do you find it difficult to follow a conversation if there is background noise (such as TV, radio, children playing)?”. HL was defined as a positive response to the hearing aid question or to both of the other questions [2]. These questions were measured at baseline between 2006 and 2010, with three follow-up assessments conducted in 2012-2013, 2014, and 2019.

For 502,017 individuals with matched sex between self-report and genetics, we excluded those missing value on loneliness (9,857) and hearing status (17,033) at baseline. Then, individuals having HL at baseline (200,753) and without hearing status at any follow-up (235,865) were also excluded. Finally, we excluded those with missing values on any covariates (5,353) and a total of 33,156 participants were included analyses. During a median follow-up period of 11.7 years, 9,432 participants (28.4%) developed HL.

***Detailed information of neuroticism.***

Neuroticism was assessed at baseline recruitment using the 12-item neuroticism scale from the Eysenck Personality Questionnaire-Revised Short Form (EPQ-R-S), with each “yes” response contributing 1 point to the total score [3]. We derived neuroticism scores using 11 of the 12 items from the scale, excluding the loneliness item [4]. The complete list of 12 questions is as follows:

|  | Questions |
| --- | --- |
| 1 | Does your mood often go up and down? |
| 2 | Do you ever feel ‘just miserable’ for no reason? |
| 3 | Are you an irritable person? |
| 4 | Are your feelings easily hurt? |
| 5 | Do you often feel ‘fed-up’? |
| 6 | Would you call yourself a nervous person? |
| 7 | Are you a worrier? |
| 8 | Would you call yourself tense or ‘highly strung’? |
| 9 | Do you worry too long after an embarrassing experience? |
| 10 | Do you suffer from ‘nerves’? |
| 11 | Do you often feel lonely? |
| 12 | Are you often troubled by feelings of guilt? |

**Table S1. Single nucleotide polymorphisms (SNPs) used to build the genetic risk score for hearing loss.**

| SNP | Chr | BP | EA | OA | EAF | Beta | SE | P value |
| --- | --- | --- | --- | --- | --- | --- | --- | --- |
| rs4660885 | 1 | 46243756 | A | G | 0.4344 | -0.007 | 9.00E-04 | 3.74E-12 |
| rs7525101 | 1 | 165109131 | T | C | 0.4424 | 0.006 | 9.00E-04 | 8.64E-11 |
| rs6545432 | 2 | 54817683 | A | G | 0.5091 | 0.007 | 9.00E-04 | 2.36E-13 |
| rs741475 | 2 | 208087139 | T | C | 0.5771 | -0.006 | 9.00E-04 | 4.02E-10 |
| rs3915060 | 3 | 121712980 | T | C | 0.7272 | -0.006 | 1.00E-03 | 3.96E-09 |
| rs72622585b | 3 | 181992315 | T | C | 0.8252 | 0.009 | 1.30E-03 | 3.41E-13 |
| rs13148153 | 4 | 17517558 | T | C | 0.1342 | 0.01 | 1.40E-03 | 2.64E-12 |
| rs323693 | 5 | 2562593 | T | C | 0.882 | -0.01 | 1.40E-03 | 1.91E-12 |
| rs1981809 | 5 | 72920029 | T | C | 0.4526 | -0.009 | 9.00E-04 | 1.36E-20 |
| rs4413512b | 5 | 73077349 | A | G | 0.5289 | -0.01 | 9.00E-04 | 1.28E-25 |
| rs13171669 | 5 | 148601243 | A | G | 0.5682 | -0.006 | 9.00E-04 | 1.61E-11 |
| rs115596275 | 6 | 32420218 | C | G | 0.0213 | 0.024 | 3.50E-03 | 2.73E-12 |
| rs7764856b | 6 | 32680640 | A | T | 0.3435 | 0.007 | 1.00E-03 | 1.10E-10 |
| rs4714678 | 6 | 43342591 | A | G | 0.4031 | -0.009 | 9.00E-04 | 7.20E-20 |
| rs9493627 | 6 | 133789728 | A | G | 0.3191 | 0.009 | 1.00E-03 | 9.56E-18 |
| rs2296508 | 6 | 158497717 | T | C | 0.4795 | -0.006 | 9.00E-04 | 4.34E-10 |
| rs11238325 | 7 | 50853151 | T | C | 0.7315 | 0.007 | 1.00E-03 | 1.97E-11 |
| rs4732339 | 7 | 138491839 | A | G | 0.5864 | 0.006 | 9.00E-04 | 6.10E-10 |
| rs150903480 | 8 | 91376248 | A | G | 0.0114 | -0.025 | 4.40E-03 | 2.70E-08 |
| rs13268718 | 8 | 141687200 | T | G | 0.5072 | -0.005 | 9.00E-04 | 7.47E-09 |
| rs2393729 | 10 | 63837016 | T | C | 0.4218 | -0.006 | 9.00E-04 | 3.07E-10 |
| rs143282422 | 10 | 73377112 | A | G | 0.0112 | 0.032 | 4.60E-03 | 6.27E-12 |
| rs1097215 | 10 | 94787804 | A | G | 0.4752 | -0.005 | 9.00E-04 | 1.11E-08 |
| rs10901863 | 10 | 126812270 | T | C | 0.2683 | 0.011 | 1.10E-03 | 9.30E-23 |
| rs7939493 | 11 | 8073610 | A | T | 0.1911 | -0.009 | 1.20E-03 | 2.47E-14 |
| rs141403654 | 11 | 47715487 | A | T | 0.9837 | -0.022 | 3.90E-03 | 2.52E-08 |
| rs147893329b | 11 | 57735006 | C | G | 0.0107 | 0.028 | 4.80E-03 | 8.17E-09 |
| rs566673 | 11 | 66401373 | T | G | 0.5339 | -0.005 | 9.00E-04 | 3.41E-08 |
| rs72963168 | 11 | 88943035 | T | C | 0.7254 | -0.009 | 1.00E-03 | 3.73E-19 |
| rs67307131 | 11 | 118480223 | T | C | 0.654 | -0.008 | 1.00E-03 | 4.62E-15 |
| rs7313797b | 12 | 109896165 | T | C | 0.5604 | -0.006 | 9.00E-04 | 7.38E-12 |
| rs35887622b | 13 | 20763620 | A | G | 0.9854 | -0.022 | 3.90E-03 | 2.59E-08 |
| rs920701 | 13 | 76417101 | T | C | 0.6357 | -0.006 | 1.00E-03 | 5.06E-11 |
| rs9517282b | 13 | 99059183 | A | C | 0.548 | -0.005 | 9.00E-04 | 3.54E-08 |
| rs1566128 | 14 | 52514981 | A | G | 0.4126 | 0.007 | 9.00E-04 | 1.42E-14 |
| rs4132250 | 15 | 89229000 | C | G | 0.778 | 0.007 | 1.10E-03 | 3.18E-11 |
| rs62033400 | 16 | 53811788 | A | G | 0.6044 | 0.005 | 9.00E-04 | 4.52E-08 |
| rs11643684 | 16 | 55490167 | T | G | 0.2031 | -0.007 | 1.10E-03 | 2.26E-09 |
| rs13337678b | 16 | 56379937 | T | C | 0.5711 | -0.005 | 9.00E-04 | 3.72E-08 |
| rs222835 | 17 | 7134129 | A | G | 0.4247 | 0.006 | 9.00E-04 | 4.81E-10 |
| rs143796236 | 17 | 79495969 | T | C | 0.0076 | 0.035 | 5.60E-03 | 2.73E-10 |
| rs11152089 | 18 | 52625943 | T | C | 0.2134 | 0.007 | 1.10E-03 | 9.24E-10 |
| rs11881070 | 19 | 2389140 | T | C | 0.2882 | -0.006 | 1.00E-03 | 5.72E-09 |
| rs12980998b | 19 | 4217510 | A | T | 0.8135 | -0.007 | 1.20E-03 | 1.02E-07 |
| rs61734651b | 20 | 61451332 | T | C | 0.0721 | 0.011 | 1.90E-03 | 8.16E-09 |
| rs5756795 | 22 | 38122122 | T | C | 0.5419 | -0.008 | 9.00E-04 | 3.65E-17 |
| rs132931 | 22 | 38487526 | A | G | 0.5869 | -0.007 | 0.0009 | 1.59E-14 |
| rs36062310 | 22 | 50988105 | A | G | 0.0427 | 0.027 | 0.0023 | 4.25E-32 |

Abbreviations: Chr, chromosome; BP, base pairs; EA, effect allele; OA, other allele; EAF, effect allele frequency; SE, standard error.

**Table S2. Hazard ratios of incident hearing loss for all predictors in the fully adjusted model.**

| **Parameter** | **HR** | **95% CI** | **P value** |
| --- | --- | --- | --- |
| Loneliness |  |  |  |
| No (Reference) | 1.00 | - | - |
| Yes | 1.24 | 1.17, 1.31 | <0.001 |
| Age | 1.09 | 1.09, 1.10 | <0.001 |
| Sex |  |  |  |
| Women (Reference) |  |  |  |
| Men | 1.39 | 1.33, 1.46 | <0.001 |
| Ethnicity |  |  |  |
| White (Reference) | 1.00 | - |  |
| Other | 0.96 | 0.84, 1.09 | 0.505 |
| Education |  |  |  |
| University degree (Reference) | 1.00 | - | - |
| No secondary education | 1.10 | 1.04, 1.17 | 0.001 |
| Secondary education | 1.08 | 1.02, 1.14 | 0.008 |
| Income levels |  |  |  |
| At least £31 000 (Reference) | 1.00 | - | - |
| Less than £31 000 | 1.25 | 1.19, 1.32 | <0.001 |
| Townsend deprivation index | 1.01 | 1.01, 1.02 | <0.001 |
| BMI, kg/m² | 1.01 | 1.01, 1.02 | <0.001 |
| Smoking status |  |  |  |
| Never (Reference) | 1.00 | - | - |
| Past | 1.12 | 1.07, 1.17 | <0.001 |
| Current | 1.18 | 1.09, 1.27 | <0.001 |
| Alcohol intake |  |  |  |
| Twice or less per week (Reference) | 1.00 | - | - |
| At least three times per week | 1.01 | 0.97, 1.05 | 0.712 |
| Physical activity |  |  |  |
| High (Reference) | 1.00 | - | - |
| Low | 0.98 | 0.93, 1.02 | 0.263 |
| Hypertension |  |  |  |
| No (Reference) | 1.00 | - | - |
| Yes | 1.05 | 1.00, 1.11 | 0.039 |
| Diabetes |  |  |  |
| No (Reference) | 1.00 | - | - |
| Yes | 1.37 | 1.27, 1.48 | <0.001 |
| Cardiovascular disease |  |  |  |
| No (Reference) | 1.00 | - | - |
| Yes | 1.25 | 1.16, 1.34 | <0.001 |
| Socially isolated |  |  |  |
| No (Reference) | 1.00 | - | - |
| Yes | 0.96 | 0.89, 1.04 | 0.287 |
| Depressed mood |  |  |  |
| Low (Reference) | 1.00 | - | - |
| High | 1.24 | 1.12, 1.37 | <0.001 |
| Use of ototoxic drugs |  |  |  |
| No (Reference) | 1.00 | - | - |
| Yes | 1.02 | 0.97, 1.07 | 0.522 |
| Genetic risk |  |  |  |
| Low (Reference) | 1.00 | - | - |
| Intermediate | 1.22 | 1.15, 1.30 | <0.001 |
| High | 1.47 | 1.37, 1.58 | <0.001 |

Abbreviations: HR, hazard ratio; CI, confidence interval; BMI, body mass index.

**Table S3. Associations of loneliness and risks of various subtypes of hearing loss.**

| **Subtypes** | **Case** | **HR** | **95% CI** | **P value** |
| --- | --- | --- | --- | --- |
| Conductive hearing loss | 357 | 1.10 | 0.84, 1.45 | 0.491 |
| Sensorineural hearing loss | 1294 | 1.23 | 1.07, 1.42 | 0.004 |
| Mixed hearing loss | 188 | 1.11 | 0.75, 1.63 | 0.604 |

Abbreviations: HR, hazard ratio; CI, confidence interval.

Note: Adjusted for age, sex, ethnicity, education, income levels, Townsend deprivation index, Body mass index, smoking status, alcohol intake, physical activity, hypertension, diabetes, cardiovascular disease, social isolation, depression, use of ototoxic drug, and genetic risk.

**Table S4. Risk of incident hearing loss according to genetic risk quintile.**

| **Genetic risk quintiles** | **HR** | **95% CI** | **P value** | **P trend** |
| --- | --- | --- | --- | --- |
| Quintile 1 (lowest) (Reference) | 1.00 | **-** | **-** | <0.001 |
| Quintile 2 | 1.17 | 1.08, 1.25 | <0.001 |  |
| Quintile 3 | 1.23 | 1.14, 1.32 | <0.001 |  |
| Quintile 4 | 1.27 | 1.19, 1.37 | <0.001 |  |
| Quintile 5 (highest) | 1.47 | 1.37, 1.58 | <0.001 |  |

Abbreviations: HR, hazard ratio; CI, confidence interval.

Note: Adjusted for age, sex, ethnicity, education, income levels, Townsend deprivation index, Body mass index, smoking status, alcohol intake, physical activity, hypertension, diabetes, cardiovascular disease, social isolation, depression, and use of ototoxic drug.

**Table S5.** **Association of loneliness and risk of incident hearing loss in the sample with imputed data on exposures.**

| **Parameter** | **HR** | **95% CI** | **P value** |
| --- | --- | --- | --- |
| Loneliness |  |  |  |
| No (Reference) | 1.00 | - | - |
| Yes | 1.19 | 1.13, 1.25 | <0.001 |
| Age | 1.09 | 1.09, 1.09 | <0.001 |
| Sex |  |  |  |
| Women (Reference) |  |  |  |
| Men | 1.42 | 1.37, 1.48 | <0.001 |
| Ethnicity |  |  |  |
| White (Reference) | 1.00 | - |  |
| Other | 0.91 | 0.83, 1.01 | 0.070 |
| Education |  |  |  |
| University degree (Reference) | 1.00 | - | - |
| No secondary education | 1.10 | 1.05, 1.15 | <0.001 |
| Secondary education | 1.08 | 1.03, 1.13 | 0.003 |
| Income levels |  |  |  |
| At least £31 000 (Reference) | 1.00 | - | - |
| Less than £31 000 | 1.21 | 1.16, 1.26 | <0.001 |
| Townsend deprivation index | 1.02 | 1.01, 1.02 | <0.001 |
| BMI, kg/m² | 1.01 | 1.01, 1.01 | <0.001 |
| Smoking status |  |  |  |
| Never (Reference) | 1.00 | - | - |
| Past | 1.14 | 1.09, 1.18 | <0.001 |
| Current | 1.15 | 1.08, 1.22 | <0.001 |
| Alcohol intake |  |  |  |
| Twice or less per week (Reference) | 1.00 | - | - |
| At least three times per week | 0.89 | 0.85, 0.92 | <0.001 |
| Physical activity |  |  |  |
| High (Reference) | 1.00 | - | - |
| Low | 0.99 | 0.95, 1.02 | 0.485 |
| Hypertension |  |  |  |
| No (Reference) | 1.00 | - | - |
| Yes | 1.06 | 1.02, 1.10 | 0.004 |
| Diabetes |  |  |  |
| No (Reference) | 1.00 | - | - |
| Yes | 1.34 | 1.26, 1.43 | <0.001 |
| Cardiovascular disease |  |  |  |
| No (Reference) | 1.00 | - | - |
| Yes | 1.22 | 1.15, 1.30 | <0.001 |
| Socially isolated |  |  |  |
| No (Reference) | 1.00 | - | - |
| Yes | 0.97 | 0.92, 1.04 | 0.414 |
| Depressed mood |  |  |  |
| Low (Reference) | 1.00 | - | - |
| High | 1.29 | 1.20, 1.40 | <0.001 |
| Use of ototoxic drugs |  |  |  |
| No (Reference) | 1.00 | - | - |
| Yes | 1.04 | 1.00, 1.08 | 0.085 |
| Genetic risk |  |  |  |
| Low (Reference) | 1.00 | - | - |
| Intermediate | 1.21 | 1.15, 1.27 | <0.001 |
| High | 1.46 | 1.38, 1.55 | <0.001 |

Abbreviations: HR, hazard ratio; CI, confidence interval; BMI, body mass index.

**Table S6. Association of loneliness and risk of incident hearing loss after excluding hearing loss events occurring within the first two years.**

| **Parameter** | **HR** | **95% CI** | **P value** |
| --- | --- | --- | --- |
| Loneliness |  |  |  |
| No (Reference) | 1.00 | - | - |
| Yes | 1.24 | 1.17, 1.32 | <0.001 |
| Age | 1.09 | 1.09, 1.10 | <0.001 |
| Sex |  |  |  |
| Women (Reference) |  |  |  |
| Men | 1.40 | 1.34, 1.47 | <0.001 |
| Ethnicity |  |  |  |
| White (Reference) | 1.00 | - |  |
| Other | 0.96 | 0.84, 1.10 | 0.552 |
| Education |  |  |  |
| University degree (Reference) | 1.00 | - | - |
| No secondary education | 1.11 | 1.04, 1.17 | <0.001 |
| Secondary education | 1.08 | 1.02, 1.15 | 0.007 |
| Income levels |  |  |  |
| At least £31 000 (Reference) | 1.00 | - | - |
| Less than £31 000 | 1.25 | 1.19, 1.31 | <0.001 |
| Townsend deprivation index | 1.01 | 1.00, 1.02 | 0.001 |
| BMI, kg/m² | 1.01 | 1.01, 1.02 | <0.001 |
| Smoking status |  |  |  |
| Never (Reference) | 1.00 | - | - |
| Past | 1.12 | 1.06, 1.17 | <0.001 |
| Current | 1.17 | 1.08, 1.26 | <0.001 |
| Alcohol intake |  |  |  |
| Twice or less per week (Reference) | 1.00 | - | - |
| At least three times per week | 1.02 | 0.97, 1.06 | 0.501 |
| Physical activity |  |  |  |
| High (Reference) | 1.00 | - | - |
| Low | 0.98 | 0.94, 1.02 | 0.370 |
| Hypertension |  |  |  |
| No (Reference) | 1.00 | - | - |
| Yes | 1.05 | 1.00, 1.11 | 0.048 |
| Diabetes |  |  |  |
| No (Reference) | 1.00 | - | - |
| Yes | 1.36 | 1.26, 1.48 | <0.001 |
| Cardiovascular disease |  |  |  |
| No (Reference) | 1.00 | - | - |
| Yes | 1.23 | 1.13, 1.33 | <0.001 |
| Socially isolated |  |  |  |
| No (Reference) | 1.00 | - | - |
| Yes | 0.97 | 0.89, 1.04 | 0.370 |
| Depressed mood |  |  |  |
| Low (Reference) | 1.00 | - | - |
| High | 1.22 | 1.10, 1.36 | <0.001 |
| Use of ototoxic drugs |  |  |  |
| No (Reference) | 1.00 | - | - |
| Yes | 1.02 | 0.96, 1.07 | 0.570 |
| Genetic risk |  |  |  |
| Low (Reference) | 1.00 | - | - |
| Intermediate | 1.23 | 1.16, 1.31 | <0.001 |
| High | 1.48 | 1.38, 1.59 | <0.001 |

Abbreviations: HR, hazard ratio; CI, confidence interval; BMI, body mass index.

**Table S7. Association of loneliness and risk of incident hearing loss** **from the Fine-Gray subdistribution Hazard Model.**

| **Parameter** | **HR** | **95% CI** | **P value** |
| --- | --- | --- | --- |
| Loneliness |  |  |  |
| No (Reference) | 1.00 | - | - |
| Yes | 1.24 | 1.17, 1.31 | <0.001 |
| Age | 1.09 | 1.08, 1.09 | <0.001 |
| Sex |  |  |  |
| Women (Reference) |  |  |  |
| Men | 1.35 | 1.29, 1.42 | <0.001 |
| Ethnicity |  |  |  |
| White (Reference) | 1.00 | - |  |
| Other | 0.97 | 0.85, 1.11 | 0.636 |
| Education |  |  |  |
| University degree (Reference) | 1.00 | - | - |
| No secondary education | 1.09 | 1.03, 1.16 | 0.002 |
| Secondary education | 1.08 | 1.02, 1.14 | 0.008 |
| Income levels |  |  |  |
| At least £31 000 (Reference) | 1.00 | - | - |
| Less than £31 000 | 1.24 | 1.18, 1.30 | <0.001 |
| Townsend deprivation index | 1.01 | 1.00, 1.02 | 0.002 |
| BMI, kg/m² | 1.01 | 1.00, 1.01 | <0.001 |
| Smoking status |  |  |  |
| Never (Reference) | 1.00 | - | - |
| Past | 1.11 | 1.05, 1.16 | <0.001 |
| Current | 1.11 | 1.02, 1.19 | 0.010 |
| Alcohol intake |  |  |  |
| Twice or less per week (Reference) | 1.00 | - | - |
| At least three times per week | 1.01 | 0.97, 1.05 | 0.660 |
| Physical activity |  |  |  |
| High (Reference) | 1.00 | - | - |
| Low | 0.97 | 0.93, 1.01 | 0.160 |
| Hypertension |  |  |  |
| No (Reference) | 1.00 | - | - |
| Yes | 1.05 | 1.00, 1.10 | 0.076 |
| Diabetes |  |  |  |
| No (Reference) | 1.00 | - | - |
| Yes | 1.30 | 1.20, 1.41 | <0.001 |
| Cardiovascular disease |  |  |  |
| No (Reference) | 1.00 | - | - |
| Yes | 1.18 | 1.10, 1.28 | <0.001 |
| Socially isolated |  |  |  |
| No (Reference) | 1.00 | - | - |
| Yes | 0.94 | 0.87, 1.01 | 0.086 |
| Depressed mood |  |  |  |
| Low (Reference) | 1.00 | - | - |
| High | 1.23 | 1.11, 1.36 | <0.001 |
| Use of ototoxic drugs |  |  |  |
| No (Reference) | 1.00 | - | - |
| Yes | 1.02 | 0.97, 1.07 | 0.410 |
| Genetic risk |  |  |  |
| Low (Reference) | 1.00 | - | - |
| Intermediate | 1.22 | 1.15, 1.30 | <0.001 |
| High | 1.47 | 1.37, 1.58 | <0.001 |

Abbreviations: HR, hazard ratio; CI, confidence interval; BMI, body mass index.

**Table S8. Association of loneliness and risk of incident self-reported hearing loss.**

| **Parameter** | **HR** | **95% CI** | **P value** |
| --- | --- | --- | --- |
| Loneliness |  |  |  |
| No (Reference) | 1.00 | - | - |
| Yes | 1.14 | 1.07, 1.21 | <0.001 |
| Age | 1.02 | 1.02, 1.03 | <0.001 |
| Sex |  |  |  |
| Women (Reference) |  |  |  |
| Men | 1.29 | 1.24, 1.35 | <0.001 |
| Ethnicity |  |  |  |
| White (Reference) | 1.00 | - |  |
| Other | 0.86 | 0.75, 0.98 | 0.025 |
| Education |  |  |  |
| University degree (Reference) | 1.00 | - | - |
| No secondary education | 0.92 | 0.87, 0.98 | 0.005 |
| Secondary education | 0.93 | 0.89, 0.97 | 0.002 |
| Income levels |  |  |  |
| At least £31 000 (Reference) | 1.00 | - | - |
| Less than £31 000 | 0.99 | 0.95, 1.04 | 0.737 |
| Townsend deprivation index | 1.01 | 1.00, 1.02 | 0.003 |
| BMI, kg/m² | 1.00 | 1.00, 1.01 | 0.813 |
| Smoking status |  |  |  |
| Never (Reference) | 1.00 | - | - |
| Past | 1.07 | 1.02, 1.12 | 0.004 |
| Current | 1.03 | 0.94, 1.12 | 0.559 |
| Alcohol intake |  |  |  |
| Twice or less per week (Reference) | 1.00 | - | - |
| At least three times per week | 1.01 | 0.96, 1.05 | 0.816 |
| Physical activity |  |  |  |
| High (Reference) | 1.00 | - | - |
| Low | 1.03 | 0.99, 1.08 | 0.133 |
| Hypertension |  |  |  |
| No (Reference) | 1.00 | - | - |
| Yes | 1.00 | 0.95, 1.05 | 0.919 |
| Diabetes |  |  |  |
| No (Reference) | 1.00 | - | - |
| Yes | 0.94 | 0.83, 1.07 | 0.372 |
| Cardiovascular disease |  |  |  |
| No (Reference) | 1.00 | - | - |
| Yes | 1.14 | 1.01, 1.28 | 0.029 |
| Socially isolated |  |  |  |
| No (Reference) | 1.00 | - | - |
| Yes | 0.98 | 0.90, 1.06 | 0.545 |
| Depressed mood |  |  |  |
| Low (Reference) | 1.00 | - | - |
| High | 1.30 | 1.15, 1.47 | <0.001 |
| Use of ototoxic drugs |  |  |  |
| No (Reference) | 1.00 | - | - |
| Yes | 1.11 | 1.06, 1.17 | <0.001 |
| Genetic risk |  |  |  |
| Low (Reference) | 1.00 | - | - |
| Intermediate | 1.24 | 1.17, 1.33 | <0.001 |
| High | 1.09 | 1.04, 1.15 | 0.001 |

Abbreviations: HR, hazard ratio; CI, confidence interval; BMI, body mass index.

**Table S9. Association of loneliness and risk of incident hearing loss** **after treating depressive mood, social isolation, and genetic risk as continuous variables.**

| **Parameter** | **HR** | **95% CI** | **P value** |
| --- | --- | --- | --- |
| Loneliness |  |  |  |
| No (Reference) | 1.00 | - | - |
| Yes | 1.18 | 1.11, 1.26 | <0.001 |
| Age | 1.09 | 1.09, 1.10 | <0.001 |
| Sex |  |  |  |
| Women (Reference) |  |  |  |
| Men | 1.41 | 1.35, 1.48 | <0.001 |
| Ethnicity |  |  |  |
| White (Reference) | 1.00 | - |  |
| Other | 0.93 | 0.82, 1.07 | 0.322 |
| Education |  |  |  |
| University degree (Reference) | 1.00 | - | - |
| No secondary education | 1.09 | 1.03, 1.16 | 0.002 |
| Secondary education | 1.07 | 1.01, 1.13 | 0.015 |
| Income levels |  |  |  |
| At least £31 000 (Reference) | 1.00 | - | - |
| Less than £31 000 | 1.23 | 1.17, 1.30 | <0.001 |
| Townsend deprivation index | 1.01 | 1.01, 1.02 | <0.001 |
| BMI, kg/m² | 1.01 | 1.00, 1.01 | <0.001 |
| Smoking status |  |  |  |
| Never (Reference) | 1.00 | - | - |
| Past | 1.14 | 1.08, 1.19 | <0.001 |
| Current | 1.19 | 1.10, 1.29 | <0.001 |
| Alcohol intake |  |  |  |
| Twice or less per week (Reference) | 1.00 | - | - |
| At least three times per week | 0.89 | 0.85, 0.94 | <0.001 |
| Physical activity |  |  |  |
| High (Reference) | 1.00 | - | - |
| Low | 0.97 | 0.93, 1.02 | 0.247 |
| Hypertension |  |  |  |
| No (Reference) | 1.00 | - | - |
| Yes | 1.07 | 1.02, 1.12 | 0.008 |
| Diabetes |  |  |  |
| No (Reference) | 1.00 | - | - |
| Yes | 1.35 | 1.24, 1.46 | <0.001 |
| Cardiovascular disease |  |  |  |
| No (Reference) | 1.00 | - | - |
| Yes | 1.24 | 1.15, 1.33 | <0.001 |
| Social isolation | 0.97 | 0.94, 1.00 | 0.055 |
| Depressed mood | 1.12 | 1.08, 1.16 | <0.001 |
| Use of ototoxic drugs |  |  |  |
| No (Reference) | 1.00 | - | - |
| Yes | 1.02 | 0.97, 1.07 | 0.517 |
| Genetic risk | 1.13 | 1.11, 1.16 | <0.001 |

Abbreviations: HR, hazard ratio; CI, confidence interval; BMI, body mass index.

**Table S10. Association of loneliness and risk of incident hearing loss with neuroticism included as an additional covariate.**

| **Parameter** | **HR** | **95% CI** | **P value** |
| --- | --- | --- | --- |
| Loneliness |  |  |  |
| No (Reference) | 1.00 | - | - |
| Yes | 1.15 | 1.08, 1.24 | <0.001 |
| Age | 1.09 | 1.09, 1.09 | <0.001 |
| Sex |  |  |  |
| Women (Reference) |  |  |  |
| Men | 1.44 | 1.37, 1.52 | <0.001 |
| Ethnicity |  |  |  |
| White (Reference) | 1.00 | - |  |
| Other | 0.9 | 0.77, 1.05 | 0.178 |
| Education |  |  |  |
| University degree (Reference) | 1.00 | - | - |
| No secondary education | 1.09 | 1.03, 1.16 | 0.005 |
| Secondary education | 1.07 | 1.00, 1.14 | 0.038 |
| Income levels |  |  |  |
| At least £31 000 (Reference) | 1.00 | - | - |
| Less than £31 000 | 1.24 | 1.17, 1.31 | <0.001 |
| Townsend deprivation index | 1.01 | 1.00, 1.02 | 0.021 |
| BMI, kg/m² | 1.01 | 1.00, 1.01 | 0.002 |
| Smoking status |  |  |  |
| Never (Reference) | 1.00 | - | - |
| Past | 1.13 | 1.08, 1.19 | <0.001 |
| Current | 1.18 | 1.09, 1.29 | <0.001 |
| Alcohol intake |  |  |  |
| Twice or less per week (Reference) | 1.00 | - | - |
| At least three times per week | 0.89 | 0.85, 0.94 | <0.001 |
| Physical activity |  |  |  |
| High (Reference) | 1.00 | - | - |
| Low | 0.97 | 0.92, 1.01 | 0.149 |
| Hypertension |  |  |  |
| No (Reference) | 1.00 | - | - |
| Yes | 1.08 | 1.02, 1.14 | 0.004 |
| Diabetes |  |  |  |
| No (Reference) | 1.00 | - | - |
| Yes | 1.37 | 1.26, 1.49 | <0.001 |
| Cardiovascular disease |  |  |  |
| No (Reference) | 1.00 | - | - |
| Yes | 1.23 | 1.13, 1.34 | <0.001 |
| Socially isolated |  |  |  |
| No (Reference) | 1.00 | - | - |
| Yes | 0.96 | 0.89, 1.05 | 0.381 |
| Depressed mood |  |  |  |
| Low (Reference) | 1.00 | - | - |
| High | 1.22 | 1.09, 1.36 | <0.001 |
| Use of ototoxic drugs |  |  |  |
| No (Reference) | 1.00 | - | - |
| Yes | 1.03 | 0.97, 1.09 | 0.337 |
| Genetic risk |  |  |  |
| Low (Reference) | 1.00 | - | - |
| Intermediate | 1.21 | 1.13, 1.29 | <0.001 |
| High | 1.47 | 1.36, 1.59 | <0.001 |
| Neuroticism | 1.02 | 1.01, 1.03 | <0.001 |

Abbreviations: HR, hazard ratio; CI, confidence interval; BMI, body mass index.

**Table S11. Baseline characteristics of participants after propensity score matching.**

|  | Not lonely |  | Lonely | SMD |
| --- | --- | --- | --- | --- |
|  | N=61678 |  | N=61678 |  |
| Age, years |  |  |  |  |
| Mean (SD) | 55.17 (8.24) |  | 55.24 (8.00) | <0.01 |
| Sex |  |  |  |  |
| Female | 37576 (60.9) |  | 37285 (60.5) | <0.01 |
| Male | 24102 (39.1) |  | 24393 (39.5) | <0.01 |
| Ethnicity |  |  |  |  |
| White | 58149 (94.3) |  | 58327 (94.6) | 0.01 |
| Other | 3529 (5.7) |  | 3351 (5.4) | 0.01 |
| Education |  |  |  |  |
| No secondary education | 21659 (35.1) |  | 21445 (34.8) | <0.01 |
| Secondary education | 20532 (33.3) |  | 20624 (33.4) | <0.01 |
| University degree | 19487 (31.6) |  | 19609 (31.8) | <0.01 |
| Income levels |  |  |  |  |
| Less than £31 000 | 35339 (57.3) |  | 35129 (57.0) | <0.01 |
| At least £31 000 | 26339 (42.7) |  | 26549 (43.0) | <0.01 |
| Townsend deprivation index |  |  |  |  |
| Mean (SD) | -0.79 (3.27) |  | -0.83 (3.25) | 0.01 |
| BMI, kg/m² |  |  |  |  |
| Mean (SD) | 27.79 (5.16) |  | 27.80 (5.19) | <0.01 |
| Smoking status |  |  |  |  |
| Never | 31849 (51.6) |  | 31989 (51.9) | <0.01 |
| Past | 21009 (34.1) |  | 21012 (34.1) | <0.01 |
| Current | 8820 (14.3) |  | 8677 (14.1) | <0.01 |
| Alcohol intake |  |  |  |  |
| Twice or less per week | 34665 (56.2) |  | 34687 (56.2) | <0.01 |
| At least three times per week | 27013 (43.8) |  | 26991 (43.8) | <0.01 |
| Physical activity |  |  |  |  |
| Low | 37397 (60.6) |  | 37319 (60.5) | <0.01 |
| High | 24281 (39.4) |  | 24359 (39.5) | <0.01 |
| Hypertension |  |  |  |  |
| No | 43636 (70.7) |  | 43490 (70.5) | <0.01 |
| Yes | 18042 (29.3) |  | 18188 (29.5) | <0.01 |
| Diabetes |  |  |  |  |
| No | 58033 (94.1) |  | 58002 (94.0) | <0.01 |
| Yes | 3645 (5.9) |  | 3676 (6.0) | <0.01 |
| Cardiovascular disease |  |  |  |  |
| No | 57802 (93.7) |  | 57783 (93.7) | <0.01 |
| Yes | 3876 (6.3) |  | 3895 (6.3) | <0.01 |
| Socially isolated |  |  |  |  |
| No | 51733 (83.9) |  | 52081 (84.4) | <0.01 |
| Yes | 9945 (16.1) |  | 9597 (15.6) | <0.01 |
| Depressed mood |  |  |  |  |
| Low | 55466 (89.9) |  | 54662 (88.6) | 0.04 |
| High | 6212 (10.1) |  | 7016 (11.4) | 0.04 |
| Use of ototoxic drugs |  |  |  |  |
| No | 42483 (68.9) |  | 42791 (69.4) | 0.01 |
| Yes | 19195 (31.1) |  | 18887 (30.6) | 0.01 |
| Genetic risk |  |  |  |  |
| Low | 12353 (20.0) |  | 12365 (20.0) | <0.01 |
| Intermediate | 37366 (60.6) |  | 37217 (60.3) | <0.01 |
| High | 11959 (19.4) |  | 12096 (19.6) | <0.01 |

Abbreviations: SMD, standardized mean difference; SD, standard deviation; BMI, body mass index.

Note: We matched participants with loneliness to those without loneliness (1:1 ratio) on a range of covariates, including age, sex, ethnicity, education, income levels, Townsend deprivation index, Body mass index, smoking status, alcohol intake, physical activity, hypertension, diabetes, cardiovascular disease, social isolation, depression, use of ototoxic drug, and genetic risk.

**Table S12. Association of loneliness and risk of incident hearing loss after propensity score matching.**

| **Loneliness** | **Case** | **HR** | **95% CI** | **P value** |
| --- | --- | --- | --- | --- |
| No (Reference) | 1255 | 1.00 | - | - |
| Yes | 1561 | 1.23 | 1.15, 1.33 | <0.001 |

Abbreviations: HR, hazard ratio; CI, confidence interval.

Note: We matched participants with loneliness to those without loneliness (1:1 ratio) on a range of covariates, including age, sex, ethnicity, education, income levels, Townsend deprivation index, Body mass index, smoking status, alcohol intake, physical activity, hypertension, diabetes, cardiovascular disease, social isolation, depression, use of ototoxic drug, and genetic risk.

**Figure S1. Flowchart of participants selection**

**Figure S2. Distribution of the genetic risk score for hearing loss**


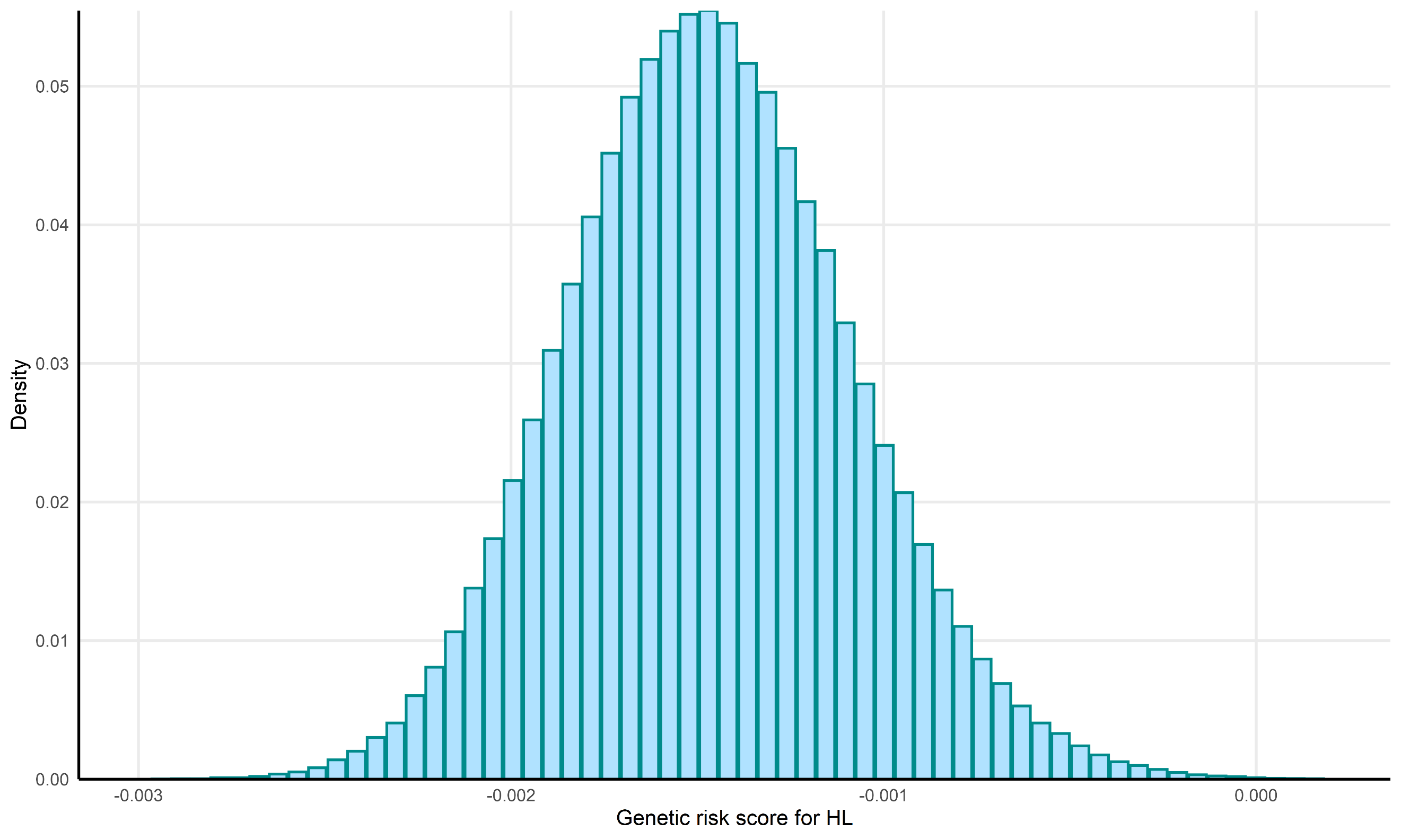


Abbreviations: HL, hearing loss.

**Figure S3. Associations of loneliness and risk of incident hearing loss across subgroups of sex, chronic diseases, social isolation, and genetic risk.**


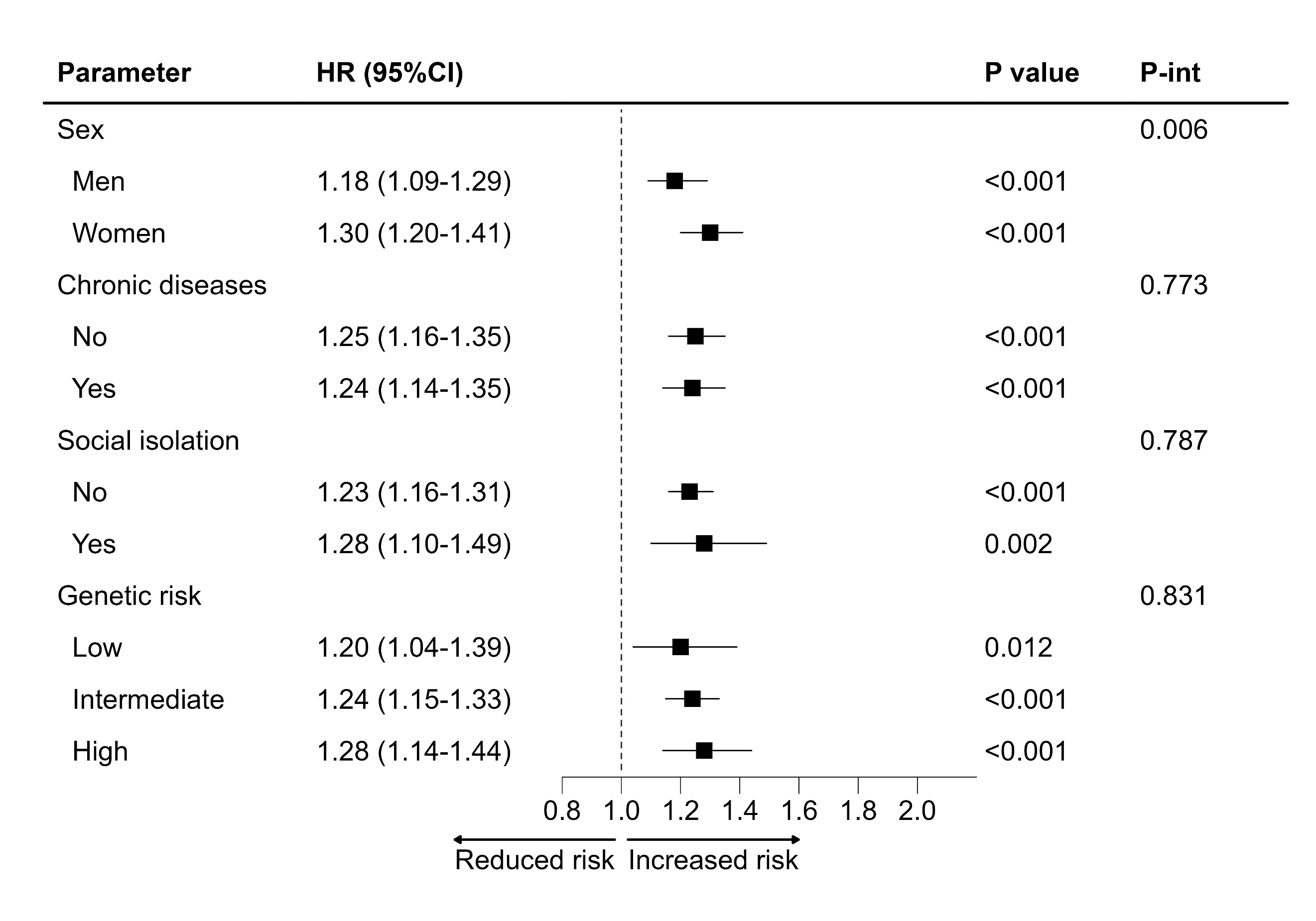


Abbreviations: HR, hazard ratio; CI, confidence interval; P-int, P for interaction.

Note: All models adjusted for age, sex, ethnicity, education, income levels, Townsend deprivation index, Body mass index, smoking status, alcohol intake, physical activity, hypertension, diabetes, cardiovascular disease, social isolation, depression, use of ototoxic drugs, and genetic risk, except for their respective stratification variables.

**References**

1. Cherny, S.S., et al., *Self-reported hearing loss questions provide a good measure for genetic studies: a polygenic risk score analysis from UK Biobank.* European Journal of Human Genetics, 2020. **28**(8): p. 1056-1065.

2. Jung, S.-H., et al., *Association between genetic risk and adherence to healthy lifestyle for developing age-related hearing loss.* BMC Medicine, 2024. **22**(1): p. 141.

3. Eysenck, S.B.G., H.J. Eysenck, and P. Barrett, *A revised version of the psychoticism scale.* Personality and Individual Differences, 1985. **6**(1): p. 21-29.

4. Shen, C., et al., *Associations of Social Isolation and Loneliness With Later Dementia.* Neurology, 2022. **99**(2): p. e164-e175.
